# RNA‐Seq of *in planta*‐expressed *Magnaporthe oryzae* genes identifies *MoSVP* as a highly expressed gene required for pathogenicity at the initial stage of infection

**DOI:** 10.1111/mpp.12869

**Published:** 2019-09-27

**Authors:** Motoki Shimizu, Yuki Nakano, Akiko Hirabuchi, Kae Yoshino, Michie Kobayashi, Kosuke Yamamoto, Ryohei Terauchi, Hiromasa Saitoh

**Affiliations:** ^1^ Division of Genomics and Breeding Iwate Biotechnology Research Center Iwate 024‐0003 Japan; ^2^ Department of Molecular Microbiology Faculty of Life Sciences Tokyo University of Agriculture Tokyo 156‐8502 Japan; ^3^ Laboratory of Crop Evolution Graduate School of Agriculture Kyoto University Kyoto 617‐0001 Japan

**Keywords:** barley, effector genes, expression profiling, gene knockout, HsbA, *Magnaporthe oryzae*, RNA‐Seq

## Abstract

The ascomycete fungus *Magnaporthe oryzae* is a hemibiotrophic pathogen that causes rice blast disease. *Magnaporthe oryzae* infects rice leaves, stems and panicles, and induces severe reductions in yield. Effector proteins secreted by *M. oryzae in planta* are thought to be involved its virulence activity. Here, using RNA‐sequencing (RNA‐Seq), we generated transcriptome data for *M. oryzae* isolate Ina168 during the initial stages of infection. We prepared samples from conidia (the inoculum) and from peeled epidermal cotyledon tissue of susceptible barley *Hordeum vulgare* ‘Nigrate’ at 12, 24, 36 and 48 hours post‐inoculation (hpi). We also generated a draft genome sequence of *M. oryzae* isolate Ina168 and used it as a reference for mapping the RNA‐Seq reads. Gene expression profiling across all stages of *M. oryzae* infection revealed 1728 putative secreted effector protein genes. We selected seven such genes that were strongly up‐regulated at 12 hpi and down‐regulated at 24 or 36 hpi and performed gene knockout analysis to determine their roles in pathogenicity. Knockout of *MoSVP*, encoding a small putative secreted protein with a hydrophobic surface binding protein A domain, resulted in a reduction in pathogenicity, suggesting that MoSVP is a novel virulence effector of *M. oryzae*.

## Introduction

The rice blast fungus *Magnaporthe oryzae* is among the most economically devastating rice pathogens worldwide (Wilson and Talbot, 2009). The rice–*M. oryzae* pathosystem is a model system for studying plant–fungal interactions due to the economic importance of rice. It is crucial to uncover details about gene‐for‐gene and protein–protein interactions between *M. oryzae* and rice in order to control this disease and develop resistant rice cultivars. During successful infection *M. oryzae* secretes a battery of proteins called effectors that help dampen host resistance and manipulate host metabolism for the benefit of the pathogen (Hogenhout *et al.*, 2009).

Transcriptomic analyses have uncovered putative proteins from *M. oryzae* that are secreted at the infection stage (Matsumura *et al.*, 2003; Saitoh *et al.*, 2012), and high‐throughput sequencing has accelerated effector gene identification. Over 40 avirulence (*AVR*) genes have been identified as involved in rice–*M. oryzae* interactions (Zhang and Xu, 2014), and increasing evidence suggests that *AVR* genes play roles in the infection of susceptible host plants. For instance, the *M. oryzae* AVR effector AVR‐Pizt interacts with the rice RING E3 ubiquitin ligase APIP6 in the host cell cytoplasm to suppress pathogen‐associated molecular pattern (PAMP)‐triggered immunity (PTI) (Park *et al.*, 2012). By contrast, only a few non‐AVR effectors have been characterized in *M. oryzae*. The best‐characterized effector is Slp1, a secreted LysM protein that suppresses host PTI by masking PAMP molecules such as chitin in the apoplast (Mentlak *et al.*, 2012). Five non‐AVR virulence effectors, including MC69, Iug6, Iug9, Bas3 and Bas4 homologue, were reported to suppress plant cell death (Dong *et al.*, 2015; Sharpee *et al.*, 2017). These effectors appear to be required for *M. oryzae* to establish biotrophy during the early stages of infection. Among these, MC69, Iug6 and Iug9 are essential for the full virulence of *M. oryzae* (Dong *et al.*, 2015; Saitoh *et al.*, 2012).

Effector genes are generally highly expressed during the early stages of infection, and whole transcriptome sequencing (RNA‐Seq) is an attractive approach for assessing differential gene expression during plant–pathogen interactions (Dong *et al.*, 2015; Kawahara *et al.*, 2012; Kobayashi *et al.*, 2017; Nguyen *et al.*, 2018; Petitot *et al.*, 2016). However, due to the low amount of fungal biomass in host leaves during the early stages of infection, pathogen transcripts account for only a small portion of the total mRNA, hampering the identification of novel pathogen transcripts. Here, we used peeled epidermal tissues of the host plant barley after *M. oryzae* inoculation to drastically increase the ratio of fungal RNA to host RNA, allowing us to perform detailed transcriptome analysis of *M. oryzae* at the early time points of infection.

## Results

### Sequencing and assembling of the genome of the *M. oryzae* isolate Ina168

We generated a draft genome sequence of *M. oryzae* isolate Ina168 as a reference for mapping RNA‐Seq reads. Paired‐end (PE) sequence reads (1.9 Gb) representing 48‐fold genome coverage were obtained using the Illumina sequencer MiSeq. Using the short reads, we reconstructed the Ina168 reference genome with the DISCOVAR *De novo* assembler (https://www.broadinstitute.org/software/discovar/blog/). The assembled genome of Ina168 consisted of 1978 contigs with a combined length of 39.6 Mb (Fig. S1 and Table S1). The N50 value and maximum length of Ina168 contigs were 163 and 920 kb, respectively. Ninety‐five percent of the genome was covered by contigs >5 kb in size.

To validate the assembled genome, we compared the assembled Ina168 genome sequence with the reference genome sequence of *M. oryzae* isolate 70‐15 (Dean *et al.*, 2005) using CEGMA software (Parra *et al.*, 2007). The Ina168 genome assemblies covered 95.2% and 97.6% completely and partially, respectively, of the 248‐conserved core eukaryotic genes (Table S2). These scores are similar to the CEGMA results for the 70‐15 reference genome sequence (Dean *et al.*, 2005). These results thus indicate that the assembled Ina168 genome sequence was of sufficient quality for mapping RNA‐Seq reads to identify genes of interest.

### RNA‐Seq of *M. oryzae*‐inoculated barley epidermal strips

To investigate the comprehensive gene expression profiles of the blast fungus at the initial stage of infection, we performed RNA‐Seq of host leaves infected by the pathogen. In a previous study in which RNA‐Seq was applied to *M. oryzae*‐infected rice leaves at 24 h post‐inoculation (hpi), 61.5–62.4% of reads were mapped to the host rice reference genome and only 0.1–0.2% to the fungal genome (Kawahara *et al.*, 2012), whereas in another study, 75.9% of reads were mapped to the host and 1.6% to the fungal genome (Dong *et al.*, 2015). To increase the representation of pathogen RNA from *M. oryzae*‐infected plant samples, we used barley cultivar Nigrate (Ngt) as the host species in our current study. *Magnaporthe oryzae*‐inoculated abaxial cotyledon epidermal strips from barley are easy to peel off for sampling (Skamnioti *et al.*, 2007), and Ngt cotyledons are highly susceptible to various isolates of *M. oryzae* and *M. grisea* (Hyon *et al.*, 2012).

**Figure 1:**
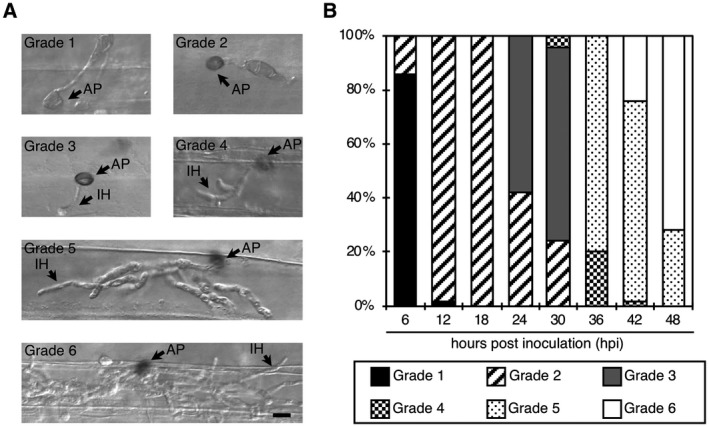
Ina168 conidia develop synchronously over 48 hpi on the abaxial epidermal cells of barley cultivar Nigrate. (A) Grades 1–6 are the stages of appressorial melanization and invasive growth. Scale bar = 10 µm. AP, appressorium; IH, invasive hypha. (B) Bar graphs showing the percentages of different grades of appressorial melanization and invasive growth at 6, 12, 18, 24, 30, 36, 42 and 48 hours post‐inoculation. Fifty appressorium‐forming conidia were counted for each time point. For a detailed description of the grade ratings, see Experimental procedures.

We sprayed a conidial suspension of Ina168 onto the abaxial sides of Ngt cotyledons and collected inoculated cotyledons at 6, 12, 18, 24, 30, 36, 42 and 48 hpi to observe infection‐related morphogenesis of Ina168 on Ngt cotyledons using microscopy. We observed 50 appressoria at each time point after Ina168 inoculation and categorized the fungal developmental stages as Grades 1 to 6 (Fig. 1A). At 6 hpi, 86% of the appressoria were not melanized (Grade 1), whereas the remaining ones showed melanization (Grade 2). At 12 and 18 hpi, most appressoria showed melanization. At 24 and 30 hpi, 56% and 76% of the melanized appressoria developed invasive hyphae (Grades 3 and 4), respectively. At 36 hpi, invasive hyphae were observed inside a single plant cell under an appressorium (Grades 4 and 5). At 42 and 48 hpi, 24% and 72% of invasive hyphae spread beyond a single cell (Grade 6), respectively (Fig. 1B). We confirmed that most conidia developed synchronously over the course of 48 hpi on the abaxial sides of Ngt cotyledons. Based on these results, we sampled the infected cotyledon tissue at 12, 24, 36 and 48 hpi for RNA extraction.

**Figure 2:**
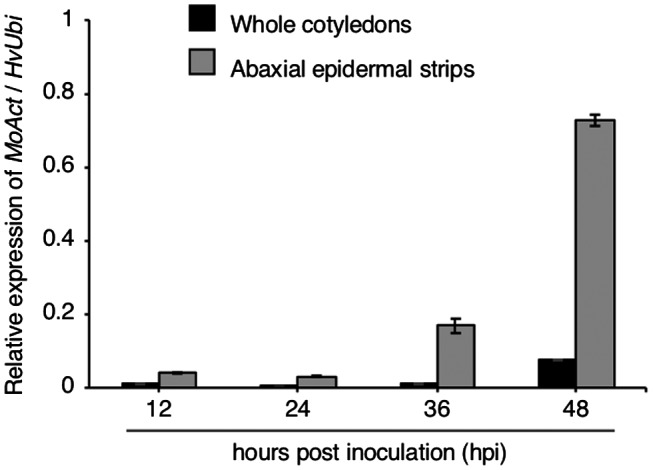
Ratio of *Magnaporthe oryzae Actin* (*MoAct*) to barley *Ubiquitin* (*HvUbi*) transcripts from Ina168‐inoculated whole leaves (black bars) and abaxial cotyledon strips containing epidermal cells (grey bars) from barley cultivar Nigrate at 12, 24, 36 and 48 hours post‐inoculation.

To examine the proportion of RNA derived from *M. oryzae* versus the host (epidermal strips from the abaxial sides of inoculated barley cotyledons), we performed quantitative reverse transcription (RT)‐PCR to detect *M. oryzae Actin* (*MoAct*) and barley *Ubiquitin* (*HvUbi*) transcripts at 12, 24, 36 and 48 hpi, and compared the results with those obtained from whole cotyledons inoculated with *M. oryzae* (Fig. 2). When we used RNA from whole Ngt cotyledons, the ratio of *MoAct* to *HvUbi* was ≤0.01:1 at 12, 24 and 36 hpi. By contrast, the ratios of *MoAct* to *HvUbi* were ~0.04:1 (12 hpi), ~0.03:1 (24 hpi) and ~0.17:1 (36 hpi) using RNA extracted from Ngt epidermal strips from the abaxial sides of cotyledons. At 48 hpi, the ratio of *MoAct* to *HvUbi* was ~0.08:1 in whole cotyledons, whereas it was ~0.73:1 in abaxial cotyledon epidermal strips. Therefore, our method using peeled barley epidermal cells dramatically increased the representation of fungal RNA compared to the use of whole cotyledons.

We inoculated Ina168 conidia onto the abaxial sides of Ngt cotyledons and peeled off the epidermal cells of inoculated foci at 12, 24, 36 and 48 hpi for RNA extraction. We also extracted RNA from conidia. We subjected the mRNA to RNA‐Seq analysis. After discarding low‐quality reads, a total of 3.8–23.4 million clean reads were generated from all samples (with three replicates). The resulting short reads were aligned against the assembled genome sequence of Ina168 with TopHat2 (Kim *et al.*, 2013) (Table S3).

### 721 *M. oryzae* genes encoding putative secreted proteins have >10‐fold differences in expression between conidia and the early infection stages

A total of 13 102 genes were predicted in the assembled genome of the Ina168 isolate of *M. oryzae*. Of these, 91% (11 893) were highly conserved (>95% DNA sequence identity) between isolates 70‐15 and Ina168, and 1209 transcripts were unique to Ina168. A total of 1728 genes encoded putative secreted proteins (see Experimental procedures). We analysed the gene expression profiles at five time points (0 [conidia inoculum], 12, 24, 36 and 48 hpi). We identified differentially expressed genes (DEGs) between the conidial stage and the four time points after Ina168 inoculation with three replicates using featureCounts/TCC with a 99% confidence level (Liao *et al.*, 2014; Sun *et al.*, 2013) (Fig. S2A). Accordingly, 468 (314 up‐ and 154 down‐regulated), 847 (686 up‐ and 161 down‐regulated), 944 (684 up‐ and 260 down‐regulated), and 996 (702 up‐ and 294 down‐regulated) DEGs encoding putative secreted proteins were identified at 12, 24, 36 and 48 hpi compared to the conidia control, respectively (Fig. S2B).

**Figure 3:**
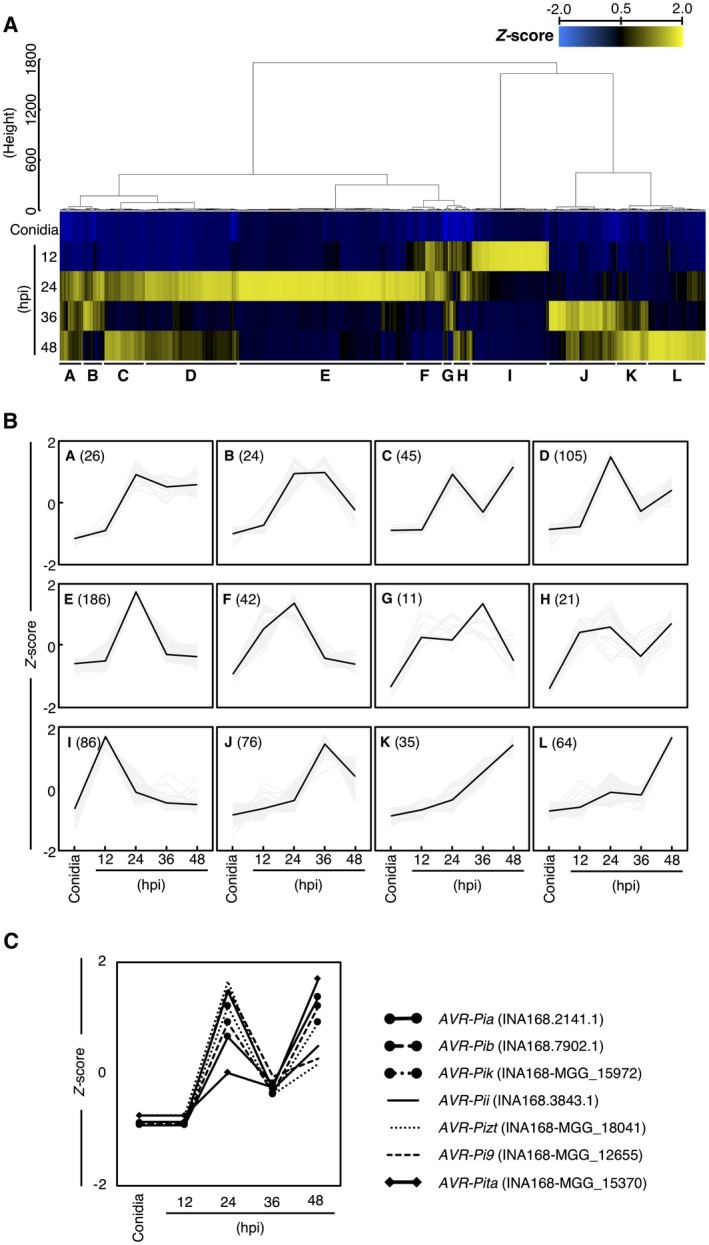
Transcriptome profile of *Magnaporthe oryzae* isolate Ina168 during barley infection. (A) Heat map showing the expression patterns of 721 DEGs encoding putative secreted proteins with >10‐fold differences in expression between conidia and the early infection stages. (B) Line plots showing the expression patterns of different gene clusters. The number of genes belonging to each cluster is shown in parentheses. (C) Expression patterns of the seven known *AVR* genes, as analysed by RNA‐Seq.

We focused on 721 DEGs encoding putative secreted proteins that were up‐regulated more than 10‐fold at any point after fungal inoculation compared to the control (conidia) to search for candidate genes for expressed effectors. We obtained the transcript per kilobase million (TPM) values for all genes at the five time points (Table S4). Based on the TPM values, we carried out hierarchical clustering of the 721 DEGs by Ward’s method using R commander (Fox, 2005), which resulted in the identification of 12 clusters (A–L) with different expression patterns (Fig. 3A,B). The largest number of genes (186 genes) belonged to cluster E; these genes were up‐regulated at 24 hpi and down‐regulated at 36 hpi. The gene encoding the apoplastic effector BAS4 (Mosquera *et al.*, 2009) was included in cluster E. Cluster I included 86 genes that were highly expressed at 12 hpi and down‐regulated at 24 hpi. Two known virulence protein genes, *Gas1* (*MAS3*: MGG_12337) and *Gas2* (*MAS1*: MGG_04202), which are specifically expressed in appressoria (Irie *et al.*, 2003; Lu *et al.*, 2005; Xue *et al.*, 2002), were included in cluster I (Table S4 and Fig. S3). The 76 genes in cluster J were up‐regulated at 36 hpi and down‐regulated at 48 hpi. This cluster included the gene encoding the apoplastic effector Slp1, which suppresses chitin‐induced host defence responses, thereby facilitating the rapid spread of the fungus within the host (Mentlak *et al.*, 2012).

We investigated the expression patterns of seven known *AVR* genes (*AVR‐Pia, ‐Pib, ‐Pii, ‐Pik, ‐Pita, ‐Pizt* and *‐Pi9*) in the Ina168 genome based on the RNA‐Seq data (Fig. 3C). *AVR‐Pia, ‐Pib* and *‐Pik* were classified into cluster C, *AVR‐Pii*, *‐Pizt* and *‐Pi9* into cluster D, and *AVR‐Pita* into cluster L (Fig. 3). The genes in all three clusters (clusters C, D and L) were up‐regulated at 24 hpi, down‐regulated at 36 hpi and up‐regulated again at 48 hpi (Fig. 3B,C).

*Cutinase 2* (*Cut2*: MGG_09100) is required by *M. oryzae* to sense the host tissue and to initiate differentiation, penetration and full virulence. *Cut2* was up‐regulated at 12 hpi and down‐regulated at 36 hpi, as revealed by RNA‐Seq (Table S4 and Fig. S3), which is consistent with its previously reported expression pattern (Skamnioti and Gurr, 2007). *Magnaporthe oryzae* isolates carrying *AVIRULENCE CONFERRING ENZYME1* (*ACE1*: MGG_12447) are specifically recognized by rice cultivars carrying the resistance gene *Pi33* (Berruyer *et al.*, 2003). *ACE1* transcripts were previously detected in trace amounts at 8 hpi, rapidly reaching a peak at 17 hpi and gradually decreasing to basal levels at 48 hpi (Fudal *et al.*, 2007). Based on our RNA‐Seq analysis, *ACE1* was not expressed at 12 hpi, highly expressed at 24 hpi and went back to basal levels at 48 hpi (Table S4 and Fig. S3). We did not perform expression analysis at 17 hpi, but our data support a previously described expression pattern of *ACE1* (Fudal *et al.*, 2007). These results indicated that our RNA‐Seq data are reliable.

### *MoSVP*, encoding a small putative secreted protein, is important for pathogenicity

To identify genes for virulence effector proteins involved in appressorial penetration, we carried out knockout (KO) analysis of putative secreted protein genes in clusters F and I that were highly up‐regulated (Fig. 3A,B). Clusters F and I included 42 and 86 putative secreted protein genes, respectively (Fig. 3B). For KO analysis, we selected seven genes that were highly expressed (>1000 TPM) at 12 hpi (Table 1). We generated Ina168 KO mutants for each of the seven genes and conducted a pathogenicity test (Table 1). To assess the virulence of each mutant, a conidial suspension of each mutant was spotted onto Ngt seedlings. Five days after inoculation, lesions had developed on seedlings inoculated with wild‐type (WT) Ina168. Among the seven mutant lines, KO of the gene INA168‐MGG_02778 led to a clear reduction in lesion development, whereas KO of the six other genes did not affect pathogenicity (Table 1). These results indicate that INA168‐MGG_02778 is required for the pathogenicity of *M. oryzae*. Therefore, we named this gene *MoSVP* (for *M. oryzae SECRETED VIRULENCE PROTEIN*).

**Table 1:** Gene knockout analysis of seven putative secreted protein genes of *Magnaporthe oryzae* isolate Ina168

Cluster	Gene ID	Accession no. (70 ‐15 isolate)	AA (Cys)	Description	TPM					Pathogenicity
					Conidia	12 hpi	24 hpi	36 hpi	48 hpi	WT 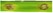
F	INA168‐MGG_02778	MGG_02778T0	180 (1)	Hypothetical	1.8	1911.8	2907.3	141.9	15.0	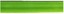
I	INA168‐MGG_12247	MGG_12247T0	220 (3)	Hypothetical	6.8	4679.9	456.5	49.0	18.8	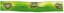
I	INA168‐MGG_02253	MGG_02253T0	330 (4)	Cell surface protein	9.7	2571.5	1533.8	85.9	17.0	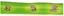
I	INA168‐MGG_05083	MGG_05083T0	146 (10)	Hypothetical	64.1	1881.5	300.1	240.4	137.3	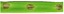
I	INA168‐MGG_09351	MGG_09351T0	430 (2)	Aspergillopepsin‐F	6.2	1650.6	431.1	49.5	51.4	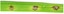
I	INA168‐MGG_01255	MGG_01255T0	406 (7)	Hypothetical	20.8	1194.0	294.4	39.8	19.2	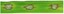
I	INA168‐MGG_16125	MGG_16125T0	200 (3)	Hypothetical	2.2	1077.6	176.7	16.6	2.8	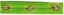

**Figure 4:**
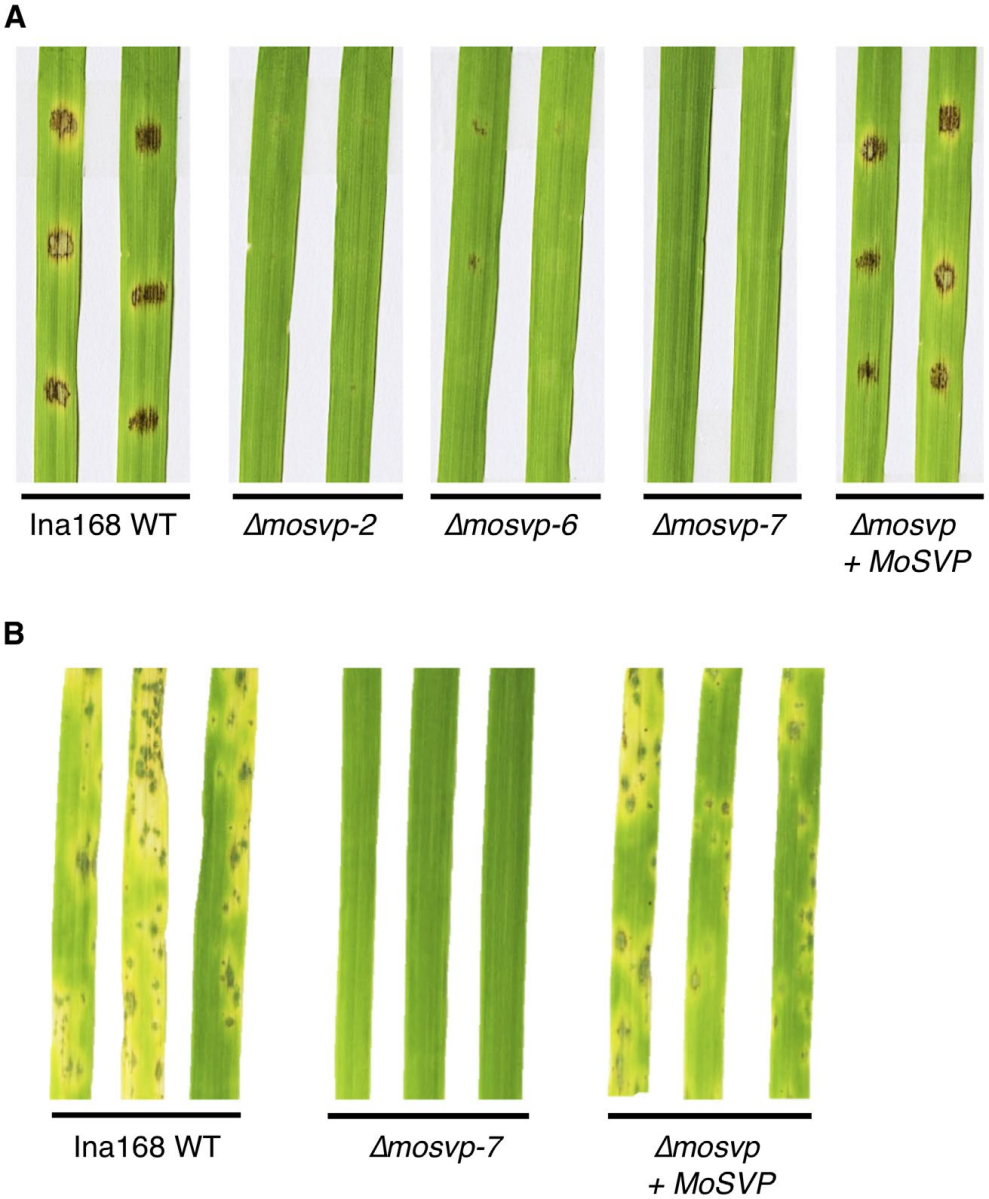
*MoSVP* is required for full virulence of *Magnaporthe oryzae* isolate Ina168 on barley cultivar Nigrate. A conidial suspension (1 × 10^4^ conidia/mL) containing Tween 20 (final concentration of 0.01%) was spotted (A) or sprayed (B) onto cotyledons. The inoculated plants were placed in a dew chamber at 27 °C for 24 h in the dark and transferred to a growth chamber with a photoperiod of 16 h. Conidial suspensions of Ina168 wild‐type (WT), *mosvp* mutant lines (∆*mosvp‐2*, *‐6* and *‐7*), and the *MoSVP*‐reintegrated strain (∆*mosvp* + *MoSVP*) were inoculated onto leaves and incubated for 5 days.

To confirm that the pathogenicity‐deficient phenotype was indeed caused by KO of *MoSVP*, we introduced an intact copy of *MoSVP* into *MoSVP*‐KO line Δ*mosvp‐7* for complementation. Ngt seedlings inoculated with the *MoSVP*‐reintegrated strain (Δ*mosvp* + *MoSVP*) developed blast disease symptoms comparable to those of seedlings inoculated with Ina168 WT on their cotyledons, as revealed by both spot and spray inoculation assays (Fig. 4).

### *MoSVP* is required for the initial stage of *M. oryzae* infection

**Figure 5:**
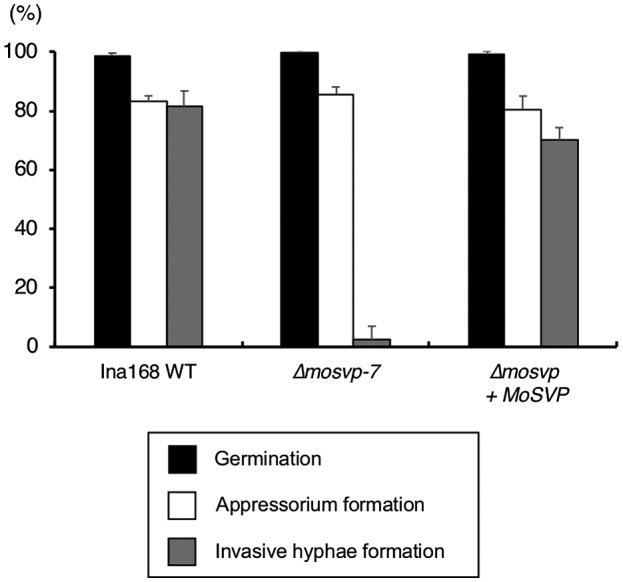
Germination, appressorium and invasive hyphae formation by Ina168 wild‐type (WT), ∆*mosvp‐7* and ∆*mosvp* + *MoSVP.* The germination ratio was calculated as the mean percentage of conidia that had germinated after 24 h on glass coverslips. The mean percentage of appressorium formation on glass coverslips among the germinated conidia is shown. Four replicates of 50 conidia were counted for each observation. The mean percentage of invasive hyphae formation at 32 hpi on barely cultivar Nigrate cotyledons from appressoria is shown. Eight replicates of 150 conidia were counted for each observation. Vertical bars indicate standard deviations.

To investigate the roles of MoSVP in various stages of *M. oryzae* infection, we observed colony growth, conidiation and the morphological characteristics of *MoSVP*‐KO compared to WT. Colony growth, colour and the production of conidia in the *mosvp* mutant (Δ*mosvp‐7*) were the same as those of Ina168 WT (Fig. S4). We then performed detailed phenotypic analysis of Δ*mosvp‐7*. Δ*mosvp‐7* was defective at forming invasive hyphae in Ngt cotyledon cells but showed normal conidial germination and appressorium formation (Fig. 5). Among the 150 appressoria of Δ*mosvp‐7*, an average of 145.5 (97.0%) failed to exhibit invasive growth in eight replicate experiments (Fig. 5), suggesting that *MoSVP* is required for the initial stage of *M. oryzae* infection. The *MoSVP*‐reintegrated strain (Δ*mosvp* + *MoSVP*) formed invasive hyphae, like those of Ina168 WT (Fig. 5). These results demonstrate that KO of *MoSVP* leads to defects in invasive growth and the development of blast symptoms in response to *M. oryzae*.

**Figure 6:**
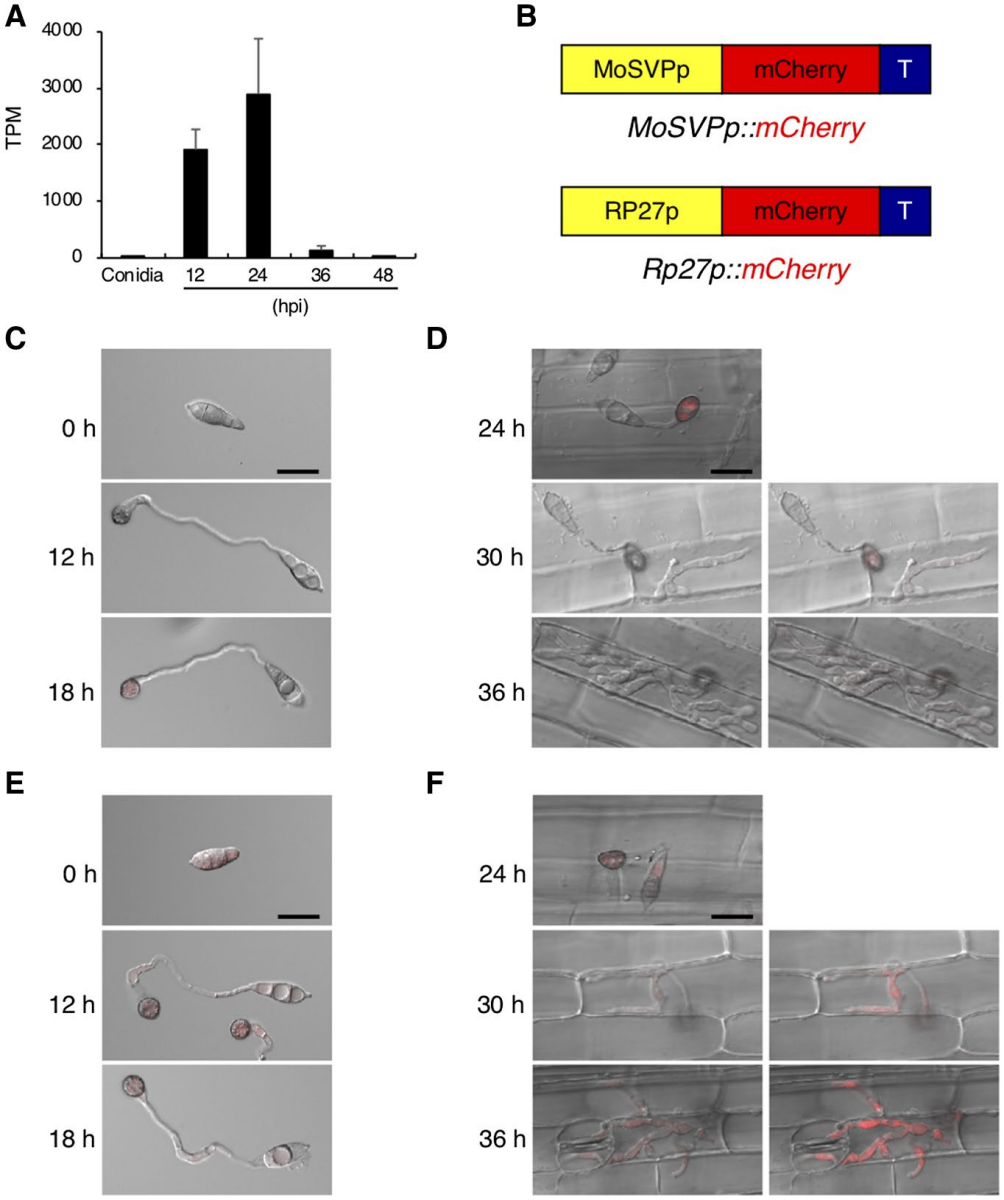
mCherry‐based promoter assay of *MoSVP* expression. (A) *MoSVP* expression pattern analysed by RNA‐Seq. (B) Schematic diagram of the mCherry protein expression constructs. (C, E) Conidia of the transgenic lines harbouring *MoSVPp::mCherry* (C) and *Rp27p::mCherry* (E) were harvested, and appressorium development and maturation were observed at 0, 12 and 18 hours post‐inoculation (hpi) on glass coverslips. Merged differential interference contrast (DIC) and mCherry (red) images are shown. Scale bar = 20 µm. (D, F) Merged DIC and mCherry images of rice leaf sheath cells infected with transformed conidia harbouring *MoSVPp::mCherry* (D) and *Rp27p::mCherry* (F) at 24, 30 and 36 hpi. Right panels show brighter images. Scale bar = 20 µm.

To investigate gene expression patterns of *MoSVP* in *M. oryzae* on glass coverslips and in leaf sheaths of the susceptible rice cultivar Moukoto, we generated *M. oryzae* carrying a reporter plasmid containing the 2.1‐kb 5ʹ upstream region of *MoSVP* fused with *mCherry* (Fig. 6B). We also generated a transgenic line harbouring a reporter plasmid containing the 0.5‐kb promoter region of *M. oryzae Ribosomal protein 27* gene (Bourett *et al.*, 2002; Li *et al.*, 2007) as a control for a constitutive expression of *mCherry* (*Rp27p::mCherry*). Strong mCherry fluorescence signals were detected in the appressoria of the *MoSVPp::mCherry* transgenic line at 18 and 24 hpi, whereas relatively weak signals were detected in the appressoria at 30 and 36 hpi and in the invasive hyphae at 30 hpi (Figs 6C,D, S5A and S6A). A control experiment with fungus transformed with *Rp27p::mCherry* showed stable expression of mCherry protein in the conidia at 0, 12, 18 and 24 hpi, in the appressoria at 12, 18, 24, 30 and 36 hpi, and in the invasive hyphae at 30 and 36 hpi (Figs 6E,F, S5B and S6B). These results indicate that *MoSVP* is highly expressed in the appressoria during the initial stage of *M. oryzae* infection, which corroborates the expression pattern of this gene detected by RNA‐Seq analysis (Fig. 6A).

### *MoSVP* is required for pathogenicity in various *M. oryzae* isolates and its homologues are found in other fungal pathogens

**Figure 7:**
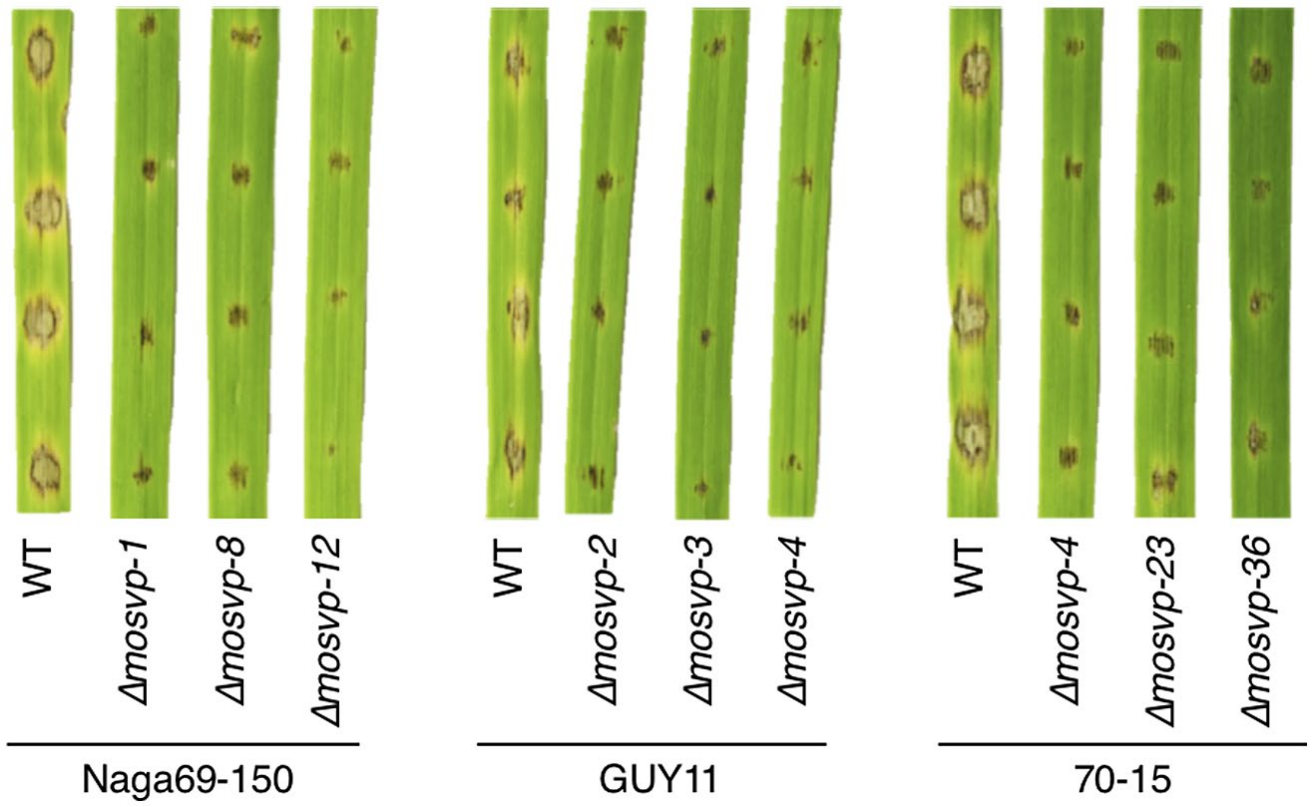
*MoSVP* is required for the pathogenicity of *Magnaporthe oryzae* isolates Naga69‐150, GUY11 and 70‐15. Conidial suspension of wild‐type (WT) isolates and three independent *mosvp* mutants per isolate were spotted onto barley cultivar Nigrate leaves and incubated for 5 days.

To investigate whether the role of MoSVP in pathogenicity is conserved in *M. oryzae*, we generated *MoSVP*‐KO mutants of three isolates, Naga69‐150, GUY11 and 70‐15, in addition to Ina168. The *MoSVP*‐KO mutants of all three isolates exhibited a clear reduction in lesion development on barley cotyledons compared to the WT (Fig. 7). These results suggest that MoSVP is required for pathogenicity in various *M. oryzae* isolates.

**Figure 8:**
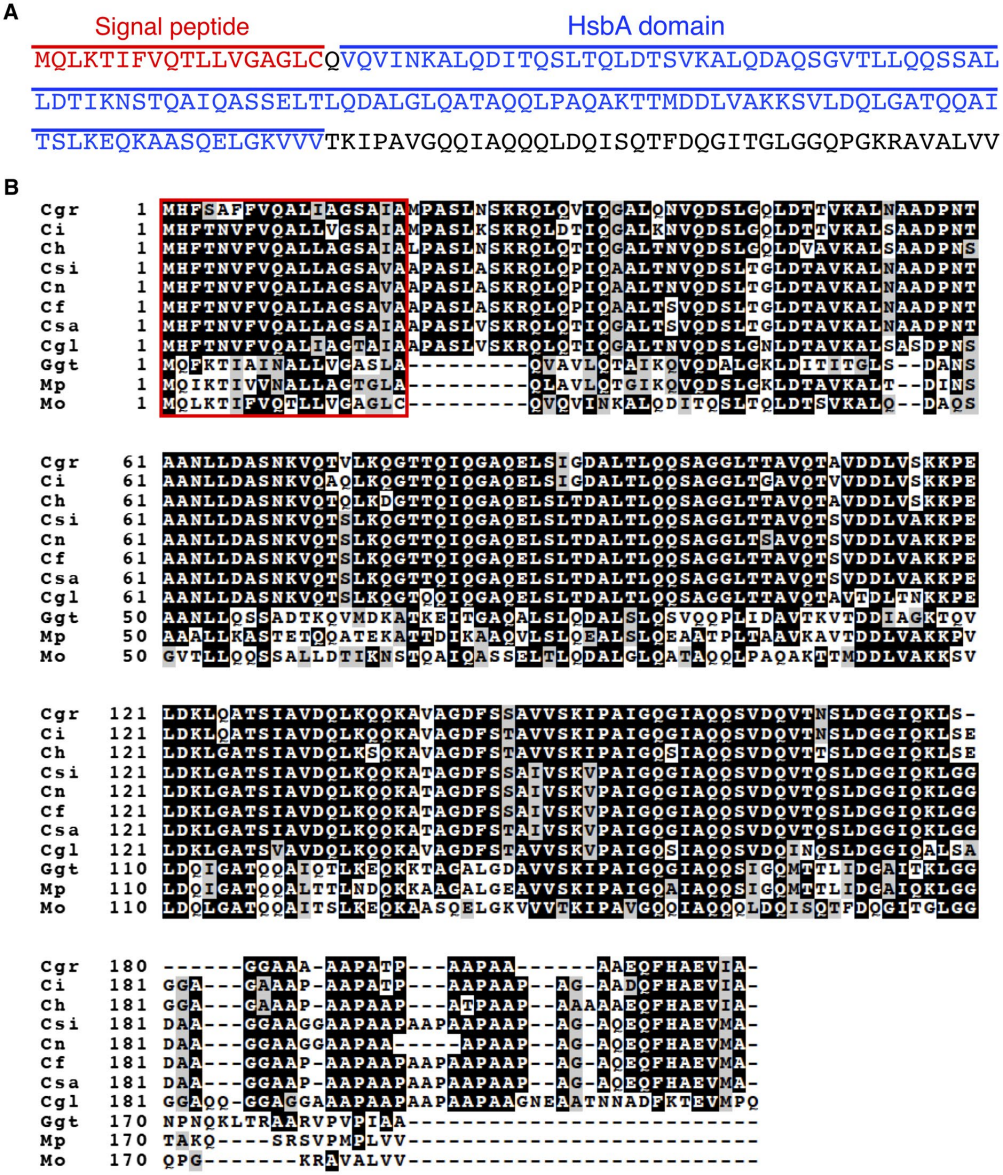
Amino acid sequence of MoSVP and sequence alignment with putative homologues from other fungal pathogens. (A) The predicted signal peptide and the HsbA domain of MoSVP are indicated in red and blue, respectively. (B) Amino acid sequence alignment of *Magnaporthe oryzae* MoSVP (Mo) with putative MoSVP homologues from *Magnaporthiopsis poae* (Mp), *Gaeumannomyces graminis* var. *tritici* (Ggt), *Colletotrichum gloeosporioides* (Cgl), *C. salicis* (Csa), *C. fioriniae* (Cf), *C. nymphaeae* (Cn), *C. simmondsii* (Csi), *C. higginsianum* (Ch), *C. incanum* (Ci) and *C. graminicola* (Cgr) generated using the ClustalW program (Tompson *et al*., 1994). Identical amino acids are indicated by white letters on a black background, similar residues are highlighted in grey and gaps introduced for alignment are indicated by hyphens. The putative signal peptide sequence is indicated by a red box.

*MoSVP* encodes a hydrophobic surface binding protein A (HsbA) domain‐containing protein with a signal peptide at its N terminus and a sequence with no known similarity to other proteins at its C terminus (Fig. 8A). HsbA from *Aspergillus oryzae* is a small protein that recruits lytic enzymes to the surfaces of hydrophobic solid materials and promotes their degradation (Ohtaki *et al.*, 2006). However, amino acid sequence alignment of MoSVP and *A. oryzae* HsbA showed that these proteins do not share high similarity (Fig. S7A).

To predict the protein structures of the HsbA domains of MoSVP (MoSVP‐HsbA) and *A. oryzae* HsbA (HsbA‐HsbA), we submitted their amino acid sequences to the PHYRE2 Protein Fold Recognition Server (http://www.sbg.bio.ic.ac.uk/~phyre2; Kelly *et al.*, 2015) and built 3D models based on the c3l1nA template. Both HsbA domains showed similar protein structures (Fig. S7B). The c3l1nA template used to build 3D models of MoSVP‐HsbA and HsbA‐HsbA is a crystal structure of one of two HsbA domains of the Mp1 protein (Mp1p) of *Talaromyces marneffei* (formerly *Penicillium marneffei*) (Liao *et al.*, 2010). *Talaromyces marneffei* is a dimorphic pathogenic fungus from Southeast Asia that primarily infects immunocompromised people. Mp1p is an immunogenic, secretory mannoprotein from *T. marneffei* that is required for virulence (Woo *et al.*, 2016).

We identified putative MoSVP homologues in other fungal pathogens, including *Magnaporthiopsis poae* (Mp), *Gaeumannomyces graminis* var. *tritici* and various *Colletotrichum* species (*C. gloeosporioides*, *C. salicis*, *C. fioriniaea*, *C. nymphaeae*, *C. simmondsii*, *C. higginsianum*, *C. incanum* and *C. graminicola*) (Fig. 8B). Although MoSVP homologues were found in other plant pathogenic fungi (Fig. 8B), their functions have not been reported. It would be interesting to determine whether these *MoSVP* homologues also contribute to the pathogenicity of various plant pathogenic fungi.

## Discussion

Biological and molecular understanding of *M. oryzae* is needed for effective control of this devastating rice pathogen. In this study, we performed RNA‐Seq to search for *M. oryzae* effectors that are expressed during the early stages of infection. To reduce the representation of host plant RNA, we used the susceptible barley cultivar Nigrate as the host, since its cotyledon epidermal cells are easily peeled off. Specifically, we extracted RNA from *M. oryzae*‐inoculated barley cotyledon epidermal strips and generated transcriptome data from conidia of *M. oryzae* isolate Ina168 before inoculation and from cotyledons at 12, 24, 36 and 48 hpi with the Ina168 isolate. RNA‐Seq analysis of the *M. oryzae* pathogen in barley epidermal cells allowed us to study the expression patterns of candidate effector genes in great detail (Fig. 3 and Table S4).

To identify novel effectors involved in the early infection processes of *M. oryzae*, we focused on putative secreted proteins. We identified 721 DEGs encoding putative secreted proteins that were up‐regulated more than 10‐fold compared to the conidia control at 12, 24, 36 or 48 hpi. These genes formed 12 clusters based on their expression patterns (Fig. 3A,B). We selected a set of seven highly expressed genes (over 1000 TPM) at 12 hpi from among the 128 predicted secreted protein genes in cluster F or I (Fig. 3B and Table 1). We reasoned that these candidate effector genes might be involved in the initial step of infection since known pathogenicity genes *Gas1* and *Gas 2* (encoding putative secreted proteins) were included in cluster I (Table S4 and Fig. S3).

KO analysis of these effector candidates in *M. oryzae* pointed to the importance of *MoSVP*, encoding the small HsbA‐domain‐containing protein (Table 1 and Figs 4, 7 and 8A). The *mosvp* mutant did not exhibit changes in saprophytic growth or conidiation on oatmeal agar medium or altered spore germination or appressorium formation on glass coverslips, but it failed to develop invasive hyphae *in planta* (Figs 5 and S4). In three previous reports, *MoSVP* (MGG_02778) was listed as a candidate effector gene based on genome‐wide transcriptional profiling of appressorium development in *M. oryzae* (Soanes *et al.*, 2012) and by RNA‐Seq analyses of mixed transcriptomes of rice interacting with *M. oryzae* (Dong *et al.*, 2015; Kawahara *et al.*, 2012). Nevertheless, gene KO analysis of *MoSVP* has not been previously performed.

*MoSVP* expression was observed in appressoria, presumably during penetration, and in primary invasive hyphae (Fig. 6). The *MoSVP* KO mutants were unable to invade plant cells to establish a compatible interaction with the host plant (Table 1, Figs 4, 5 and 7). However, other phases of infection‐related development, such as conidial germination and appressorium formation, were unaffected in the *mosvp* mutant (Fig. 5).

Two hydrophobins, MPG1 and MHP1, from *M. oryzae* are known to be required for full pathogenicity of *M. oryzae*. The class I hydrophobin protein MPG1 (MGG_10315) confers surface hydrophobicity to fungi by forming a spore rodlet layer and it is also present in appressoria (Talbot *et al.*, 1993). Deletion of *MPG1* results in an easily wettable phenotype (Talbot *et al.*, 1993). The class II hydrophobin gene *MHP1* (MGG_01173) plays essential roles in surface hydrophobicity and infection‐related fungal development (Kim *et al.*, 2005). The *mhp1* mutants show a detergent wettable phenotype, but their wettability with water is not altered (Kim *et al.*, 2005). Fungal colonies of *mosvp* mutants on oatmeal agar medium did not show an easily wettable phenotype when incubated with 10‐µL droplets of distilled water or 0.01% (v/v) Tween 20 in distilled water (data not shown). *MoSVP* was exclusively expressed during appressorial penetration and initial invasive growth (Fig. 6), suggesting that it might not affect the hydrophobicity of the surfaces of aerial hyphae. Future studies should investigate the localization of MoSVP to determine whether it is indeed secreted and to examine its role in adhesion.

MoSVP, MHP1 and MPG1 have signal peptides at their N termini and contain different hydrophobic domains, i.e. HsbA, Hydrophobin‐2 and Hydrophobins, respectively (Fig. S8A–C). In addition, the amino acid sequence similarities among these proteins are quite low (Fig. S8D). The *mhp1* mutants exhibit pleiotropic effects in terms of fungal morphogenesis, including reduced conidiation, conidial germination, appressorium development and infectious growth in host cells (Kim *et al.*, 2005). The *mpg1* mutants show reduced appressorium formation and conidiation (Talbot *et al.*, 1996). By contrast, in this study the *mosvp* mutants were not affected in saprophytic growth, conidiation or appressorium formation (Figs 5 and S4), but they showed severely reduced pathogenicity (Table 1 and Figs 4 and 7), like mutants of the previously identified *M. oryzae* effector MC69 (Saitoh *et al.*, 2012).

As a hemibiotrophic fungal pathogen, *M. oryzae* requires a living host during the initial stages of infection (biotrophic phase), followed by host cell death leading to lesion formation (necrotrophic phase). Effector screens for suppressors of plant cell death were previously carried out to identify *M. oryzae* effectors that suppress host cell death during the biotrophic phase of infection (Dong *et al.*, 2015; Sharpee *et al.*, 2017). Additional work is needed to analyse the roles of MoSVP and the other effector candidates that are highly expressed during the initial stages of *M. oryzae* infection (as revealed by RNA‐Seq) in PTI and the suppression of cell death.

Clustering analysis of the expression patterns of putative *M. oryzae* secreted protein genes revealed that most known *AVR*s share similar expression patterns (clusters C, D and L) during the early stages of infection (Fig. 3). This observation provides us with a potential approach for identifying novel *AVR*s by focusing on putative secreted protein genes belonging to these three clusters (clusters C, D and L in Fig. 3).

## Experimental Procedures

### Fungal isolate, media and transformation

All *M. oryzae* isolates used in this study were stored at the Iwate Biotechnology Research Center, Japan. The fungal isolates used were the WT isolates Ina168, Naga69‐150, GUY11 and 70‐15. The *M. oryzae* isolates were grown on oatmeal agar medium. To obtain protoplasts, *M. oryzae* hyphae were incubated for 3 days in 200 mL of YG medium (5 g of yeast extract and 20 g of glucose per litre). Protoplast preparation and transformation were performed as described previously (Takano *et al.*, 2001). Hygromycin‐ or bialaphos‐resistant transformants were selected on plates containing 300 µg/mL of hygromycin B (Wako Pure Chemicals, Japan) or 250 µg/mL of bialaphos (Wako Pure Chemicals), respectively.

### Genome sequencing and assembling

Genomic DNA was extracted from Ina168 mycelia using a NucleoSpin Plant II Kit (Macherey Nagel Co, Düren, Germany). Libraries for PE short reads were constructed using an Illumina TruSeq DNA LT Sample Prep Kit (Illumina, CA, USA). The PE library was sequenced on the Illumina MiSeq platform. The short reads of sequence data (48‐hold coverage) were assembled into genome sequences using the DISCOVAR *De novo* assembler (https://www.broadinstitute.org/software/discovar/blog/).

### Microscopy observation of fungal infection‐related morphogenesis on barley leaves

A conidial suspension (1 × 10^4^ conidia/mL) containing Tween 20 (final concentration of 0.01%) was sprayed onto the abaxial sides of the cotyledons of susceptible barley cultivar Nigrate (Ngt). The inoculated plants were placed in a dew chamber at 27 °C in the dark and harvested at 6, 12, 18, 24, 30, 32, 36 and 48 hpi. The harvested leaves were decolorized in 95% (overnight) and 75% (1–2 days) ethanol prior to microscopy. Appressorium melanization and the invasive growth ratings of the 50 appressorium‐forming conidia were scored. Appressorium melanization and invasive growth were classified into six grades: Grade 1, nonmelanized appressoria; Grade 2, melanized appressoria; Grade 3, primary invasive hyphae; Grade 4, invasive hyphae with two or three branches, Grade 5, invasive hyphae with more than three branches within a single cell; Grade 6, invasive hyphae spread into more than one cell (Fig. 1A).

### RNA extraction, quantitative RT‐PCR and RNA‐Seq

A conidial suspension (3 × 10^5^ conidia/mL) of Ina168 containing Tween 20 (final concentration of 0.01%) was sprayed onto the abaxial sides of Ngt cotyledons. The inoculated plants were placed in a dew chamber at 27 °C in the dark and harvested at 12, 24, 36 and 48 hpi. Abaxial epidermal strips were peeled, immediately frozen in liquid nitrogen and stored at −80 °C. Total RNA was extracted from peeled abaxial epidermal strips using an SV Total RNA Isolation System (Promega, WI, USA) and used for quantitative RT‐PCR (qRT‐PCR) and RNA‐Seq analysis. Total RNA for RNA‐Seq analysis was prepared from conidial suspensions in a similar manner. cDNA was synthesized from 500 ng total RNA using a PrimeScript RT Reagent Kit (Takara Bio, Otsu, Japan). The qRT‐PCR was performed using a StepOne Real‐time PCR Instrument (Applied Biosystems, CA, USA) with 10‐µL reaction mixtures containing 0.5 µL cDNA, 5 µL of KAPA SYBR FAST Universal 2 × qPCR Master Mix (Kapa Biosystems, MA, USA), 0.3 µL of each gene‐specific primer (0.1 mm) and 1.9 µL of ddH_2_O under the following reaction conditions: 95 °C for 20 s, followed by 40 cycles of denaturation at 95 °C for 3 s and annealing and extension at 60 °C for 30 s. Finally, melt curve analyses (from 60 to 95 °C) were included at the end of the cycles to ensure the consistency of the amplified products. Comparative Ct (ΔΔCt) was used to calculate the expression of the *M. oryzae Actin* gene (*MoAct*) relative to that of the barley (*Hordeum vulgare*) *Ubiquitin* gene (*HvUbi*) as an internal control. The data presented are the average and standard deviations from three experimental replications. The primers used for qRT‐PCR are listed in Table S5. For RNA‐Seq, 2 µg of total RNA was used to construct cDNA libraries using a TruSeq RNA Sample Prep Kit v. 2 (Illumina, CA, USA). The libraries were subjected to SE sequencing with 75 cycles on the NextSeq 500 platform. The sequence reads were filtered for quality using PRINSEQ (Schmieder and Edwards, 2011).

### Prediction of genes and effectors

Published reference cDNA sequences of *M. oryzae* isolate 70‐15 (Dean *et al.*, 2005) were aligned to the Ina168 genome using Exonerate (Slater and Birney, 2005) and a GTF file was generated. Reference‐guided assembly of transcripts was carried out; RNA‐Seq short reads from all samples were aligned to the Ina168 reference genome using TopHat2 and information from the GTF file generated by Exonerate was combined using StringTie (Kim *et al.*, 2013; Pertea *et al.*, 2015) (method is shown in Fig. S9). Putative secreted proteins (based on the presence of signal peptides) were predicted using SignalP v. 4.1 (Nielsen *et al.*, 1997).

### Plasmid construction

All primers used in this study are listed in Table S5. To generate the gene disruption vector pGDMoSVP, a 4.1‐kb fragment containing the 5′ flanking region of *MoSVP* was amplified by PCR with primers MoSVPko5fwd and MoSVPko5rev developed by In‐Fusion® HD Cloning (Clontech, Madison, WI, USA). The amplified product was cloned into pCB1636 (Sweigard *et al.*, 1997) containing a hygromycin resistance gene that had been linearized by digestion with *Kpn*I, generating pGD5MoSVP. A 4.0‐kb fragment containing the 3′ flanking region of *MoSVP* was amplified by PCR with primers MoSVPko3fwd and MoSVPko3rev developed by In‐Fusion® HD Cloning. The amplified product was cloned into pGD5MoSVP that had been linearized by inverse PCR using primers pCB1636iv1fwd and pCB1636iv1rev, generating pGDMoSVP. Disruption vectors of the three genes INA‐MGG_02253, INA‐MGG_09351 and INA‐MGG_16125 were generated in the same manner as pGDMoSVP construction. To generate disruption vectors of the three genes INA‐MGG_12247, INA‐MGG_05083 and INA‐MGG_01255, the 5′ flanking region was amplified by PCR using the specific primers for each gene developed by In‐Fusion® HD Cloning. The amplified product was cloned into pCB1636 (Sweigard *et al.*, 1997) containing a hygromycin resistance gene that had been linearized by inverse PCR using primers pCB1636iv2fwd and pCB1636iv2rev. The 3′ flanking region was amplified using PCR and the specific primers for each gene developed by In‐Fusion® HD Cloning. The amplified product was cloned into a plasmid containing the 5′ flanking region that had been linearized by inverse PCR using primers pCB1636iv1fwd and pCB1636iv1rev. For the complementation assay of the *mosvp* mutant with MoSVP, a 5.5‐kb fragment containing *MoSVP* was amplified with primers MoSVP_UL and MoSVP_U. The amplified product was cloned into pCB1531 (Sweigard *et al.*, 1997) containing a bialaphos resistance gene that had been linearized by inverse PCR using primers pCB1636iv2fwd and pCB1636iv2rev developed by In‐Fusion® HD Cloning, generating pCB1531‐MoSVP. To construct pCB‐MoSVPp‐mCherry, a 2.1‐kb fragment containing the 5′ flanking region of *MoSVP* was amplified by PCR using primers MoSVP‐U1 and MoSVP‐L0 developed by In‐Fusion® HD Cloning. The amplified product was cloned into pCB‐Ppwl2‐mCherry‐stop (Saitoh *et al.*, 2012) that had been linearized by digestion with *Not*I and *Xba*I, generating pCB‐MoSVPp‐mCherry (Fig. 6B). To construct pCB‐Rp27p‐mCherry, a 0.5‐kb fragment containing the 5′ flanking region of *Rp27* (Bourett *et al.*, 2002) was amplified by PCR using primers Rp27‐U1 and Rp27‐L0. The amplified product was digested with *Not*I and *Xba*I and replaced with the *Not*I/*Xba*I fragment of the *PWL2* promoter in pCB‐Ppwl2‐mCherry‐stop (Saitoh *et al.*, 2012), generating pCB‐Rp27p‐mCherry (Fig. 6B).

### Pathogenicity assays

A conidial suspension (1 × 10^4^ conidia/mL) containing Tween 20 (final concentration of 0.01%) was spotted or sprayed onto Ngt cotyledons 14 days after sowing. The inoculated plants were placed in a dew chamber at 27 °C for 24 h in the dark and transferred to a growth chamber with a photoperiod of 16 h.

### Confocal laser scanning microscopy

Germinated conidia of isolate Naga69‐150 harbouring *MoSVPp::mCherry* and *Rp27p::mCherry* were observed on glass coverslips. The rice leaf sheath inoculation assay was performed as described previously (Namai *et al.*, 1996). Appressorium‐forming conidia and invading hyphae of the transformants were observed in epidermal cells in the leaf sheaths of susceptible rice cultivar Moukoto. mCherry fluorescence (red) and differential interference contrast (DIC) images were observed under a Nikon A1R + Ni‐E confocal laser scanning microscope (Nikon, Tokyo, Japan) equipped with a LU‐N4/N4S 4‐laser unit and an Apo ×40/1.25 WI objective lens. Samples were mounted in water under glass coverslips and excited with a 561 nm laser. A DM405/488/561/640 diachronic mirror and BA570‐620 emission filter were used for observation. The pinhole setting was 24.3 µm for the merged DIC and mCherry images except for the right panels of Fig. 6D and F (pinhole setting 71.5 µm).

## Supporting information


**Fig. S1** Flow chart of *Magnaporthe oryzae *isolate Ina168 genome assembly carried out by DISCOVAR *De novo *(https://www.broadinstitute.org/software/discovar/blog/). PE, paired end.

**Fig. S2** Differentially expressed genes (DEGs) at 12, 24, 36, and 48 hours post‐inoculation (hpi) compared to 0 hpi (Conidia). (A) Number of up‐ or downregulated genes at the four time points after *Magnaporthe* *oryzae* inoculation compared to conidia. (B) Number of up‐ or down‐regulated genes encoding putative secreted proteins at the four time points after *M*. *oryzae* inoculation compared to conidia.

**Fig. S3** Expression patterns of *Gas1*, *Gas2*, *Cut2*, and *ACE1*. Transcript per kilobase million (TPM) values of the four genes at the five time points (0 [conidia inoculum], 12, 24, 36, and 48 hours post‐inoculation) were obtained by RNA‐Seq.

**Fig. S4** Colony growth and conidiation of Ina168 wild‐type (WT), an *mosvp* mutant (∆*mosvp‐7*), and the *MoSVP*‐reintegrated strain (∆*mosvp *+ *MoSVP*). (A) Colony colour and aerial hyphae production were identical among the three strains. Photographs were taken 7 days after incubation on oatmeal agar. (B) Growth and conidiation of the three strains. Mean values of colony diameter (cm) were measured after 7 days of growth on oatmeal agar. Mean values were calculated from four replicates. Conidiogenesis was assessed in four replicate experiments. Means are expressed as the number of conidia × 10^4^ of conidial suspension/cm^2^ of culture.

**Fig. S5** mCherry‐based promoter assay of *MoSVP* and *Rp27* expressions on glass coverslips. (A,B) Conidia of the transgenic lines harbouring *MoSVPp::mCherry *(A) and *Rp27p::mCherry *(B) were harvested, and appressorium development and maturation were observed at 0, 12 and 18 hours post‐inoculation (hpi) on glass coverslips. Merged differential interference contrast (DIC and mCherry (Red) images are shown in left panels. mCherry images are shown in middle panels. Green arrows in the panels show line scans used to generate corresponding fluorescence intensity distribution graphs (right panels). Pinhole setting is 24.3 µm. Scale bar = 20 µm.

**Fig. S6** mCherry‐based promoter assay of *MoSVP* and *Rp27* expression in rice leaf sheath cells. (A,B) Conidia of the transgenic lines harbouring *MoSVPp::mCherry *(A) and *Rp27p::mCherry *(B) were harvested, and appressorial penetration and invasive growth were observed at 24, 30 and 36 hours post‐inoculation (hpi) in rice leaf sheath cells. Merged differential interference contrast (DIC) and mCherry (Red) images are shown in left panels. mCherry images are shown in middle panels. Green arrows in the panels show line scans used to generate corresponding fluorescence intensity distribution graphs (right panels). Pinhole setting is 24.3 µm. Scale bar = 20 µm.

**Fig. S7** HsbA domains of MoSVP (MoSVP‐HsbA) and *Aspergillus oryzae *HsbA (HsbA‐HsbA) share low amino acid sequence similarity but have similar predicted protein structures. (A) Sequence alignment of MoSVP and *A. oryzae *HsbA. Amino acid sequences were aligned using ClustalW (Tompson *et al*., 1994). The predicted signal peptide and HsbA domains of MoSVP and *A. oryzae *HsbA are outlined in red and blue, respectively. Identical amino acids are indicated by white letters on a black background. Similar residues are highlighted in grey. Gaps introduced for alignment are indicated by hyphens. (B) Topology diagrams showing that MoSVP‐HsbA and HsbA‐HsbA possess the same fold. The ribbon diagrams were generated using PyMOL (http://www.pymol.org).

**Fig. S8** Amino acid sequences and sequence alignment of MoSVP and *Magnaporthe oryzae *hydrophobins MPG1 and MHP1. (A) The predicted signal peptide and HsbA domain of MoSVP are indicated in red and blue, respectively. (B) The predicted signal peptide and Hydrophobin_2 domain of MHP1 are indicated in red and purple, respectively. (C) The predicted signal peptide and hydrophobins domain of MPG1 are indicated in red and green, respectively. (D) Sequence alignment among MoSVP, MHP1 and MPG1. Amino acid sequences were aligned using ClustalW (Tompson *et al*., 1994). Identical amino acids are indicated by white letters on a black background. Similar residues are highlighted in grey. Gaps introduced for alignment are indicated by hyphens.

**Fig. S9** Schematic diagram of the annotation pipeline, including the input/output (grey, dashed boxes) and software (orange, solid line boxes).

**Table S1** Assembly metrics of the reference genome of *Magnaporthe oryzae *isolate Ina168.

**Table S2** Coverage of conserved eukaryotic genes in *Magnaporthe oryzae *isolate Ina168 and 70‐15 genome sequences analysed by CEGMA.

**Table S3** Summary of RNA‐Seq reads and mapping statistics.

**Table S4** List of transcipt per kilobase million (TPM) values for all detected fungal genes in *Magnaporthe oryzae* isolate Ina168.

**Table S5** Primers used for qRT‐PCR and plasmid construction.

## Data Availability

The datasets supporting the conclusions of this article are available in the DDBJ/EMBL/GenBank databases at https://www.ncbi.nlm.nih.gov/. The BioProject Accession number for this study is PRJDB8014. Sequence data for MoSVP and the putative homologues mentioned in this article can be found in the GenBank/EMBL data libraries under accession numbers MGG_02778 (Mo), KLU83140 (Mp), XP_009227853 (Ggt), QB56162 (Cgl), KXH42038 (Csa), EXF84635 (Cf), KXH37691 (Cn), KXH30155 (Csi), XP_018152909 (Ch), KZL79520 (Ci) and XP_008100613 (Cgr).
