# Supplementary figures and images for: RNA‐Seq of in planta‐expressed Magnaporthe oryzae genes identifies MoSVP as a highly expressed gene required for pathogenicity at the initial stage of infection
Source: Mol Plant Pathol. 2019 Sep 27;20(12):1682–95. doi: 10.1111/mpp.12869 (PMC6859710; doi:10.1111/mpp.12869)

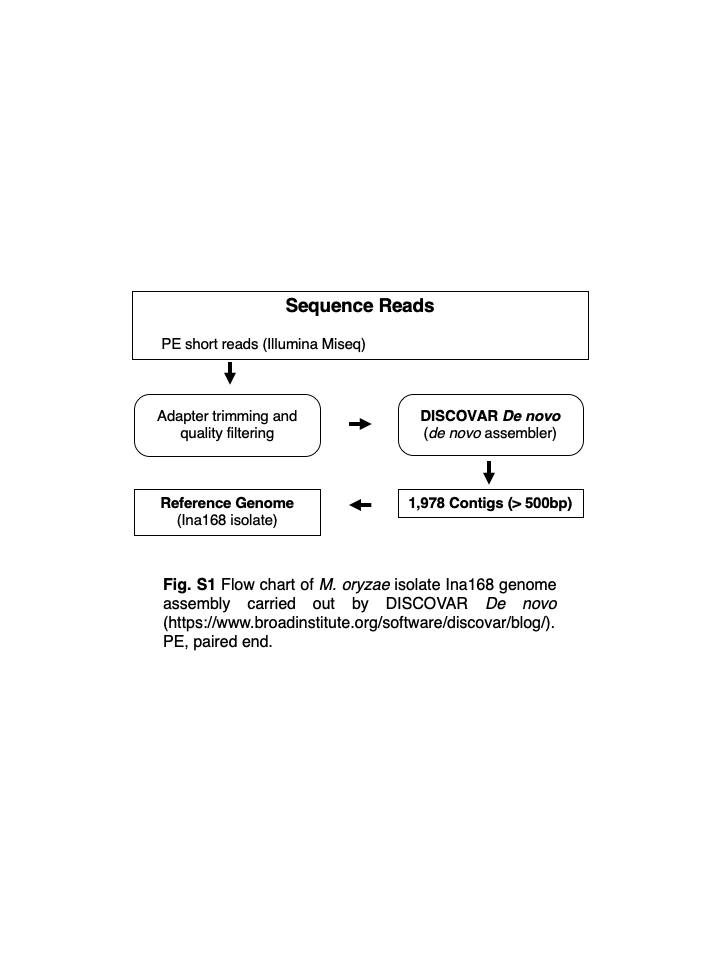

Supplement: Supplementary file 1 — Fig. S1 Flow chart of Magnaporthe oryzae isolate Ina168 genome assembly carried out by DISCOVAR De novo (https://www.broadinstitute.org/software/discovar/blog/). PE, paired end. [file MPP-20-1682-s001.tiff]

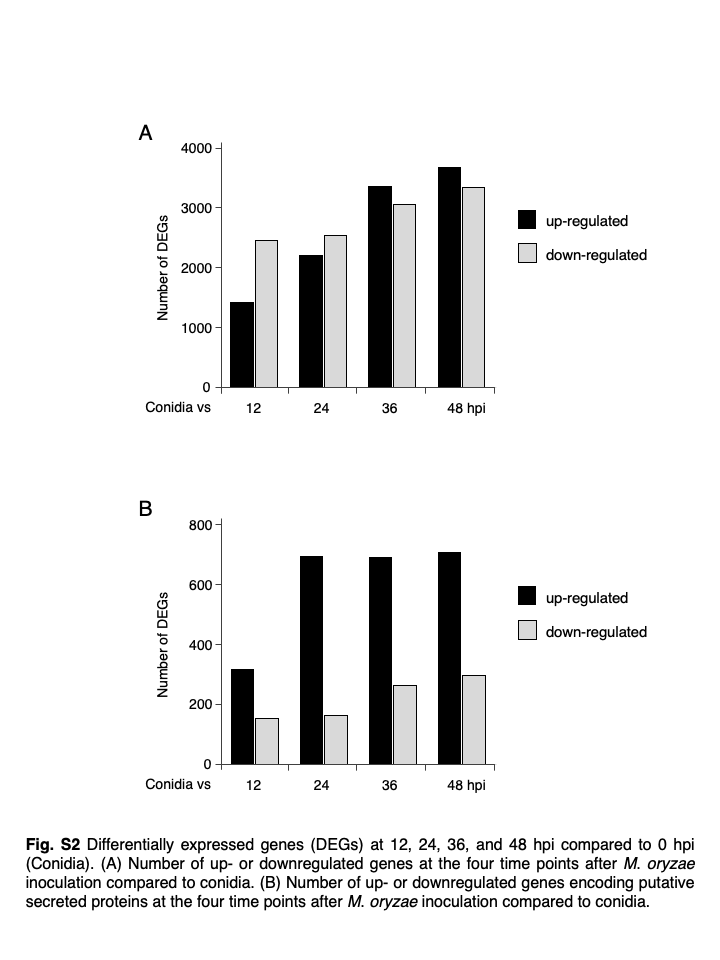

Supplement: Supplementary file 2 — Fig. S2 Differentially expressed genes (DEGs) at 12, 24, 36, and 48 hours post‐inoculation (hpi) compared to 0 hpi (Conidia). (A) Number of up‐ or downregulated genes at the four time points after Magnaporthe oryzae inoculation compared to conidia. (B) Number of up‐ or down‐regulated genes encoding putative secreted proteins at the four time points after M. oryzae inoculation compared to conidia. [file MPP-20-1682-s002.tiff]

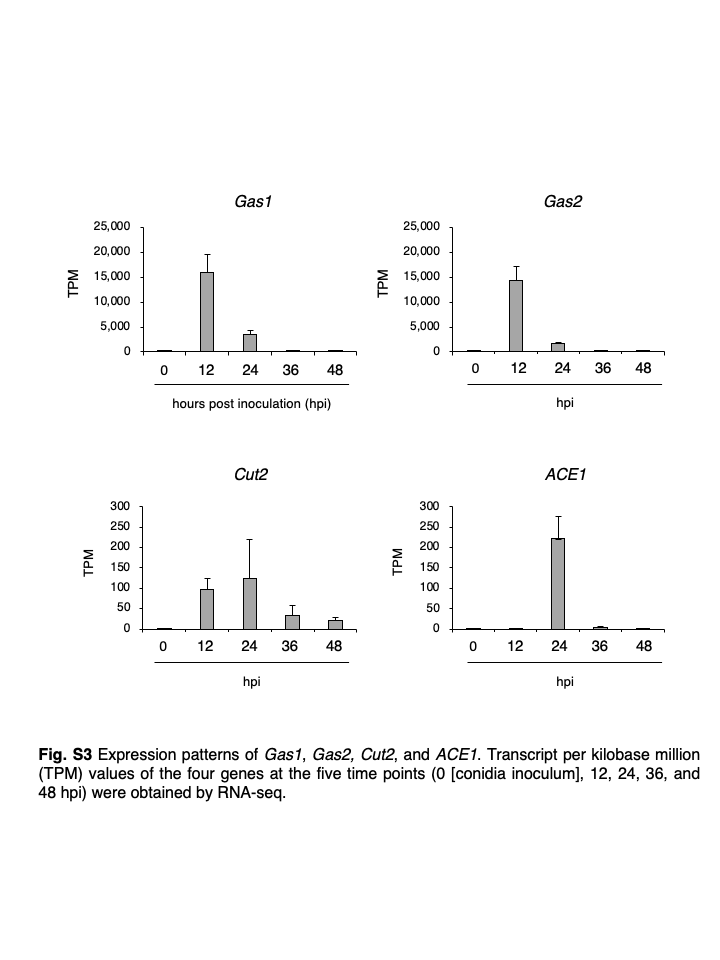

Supplement: Supplementary file 3 — Fig. S3 Expression patterns of Gas1, Gas2, Cut2, and ACE1. Transcript per kilobase million (TPM) values of the four genes at the five time points (0 [conidia inoculum], 12, 24, 36, and 48 hours post‐inoculation) were obtained by RNA‐Seq. [file MPP-20-1682-s003.tiff]

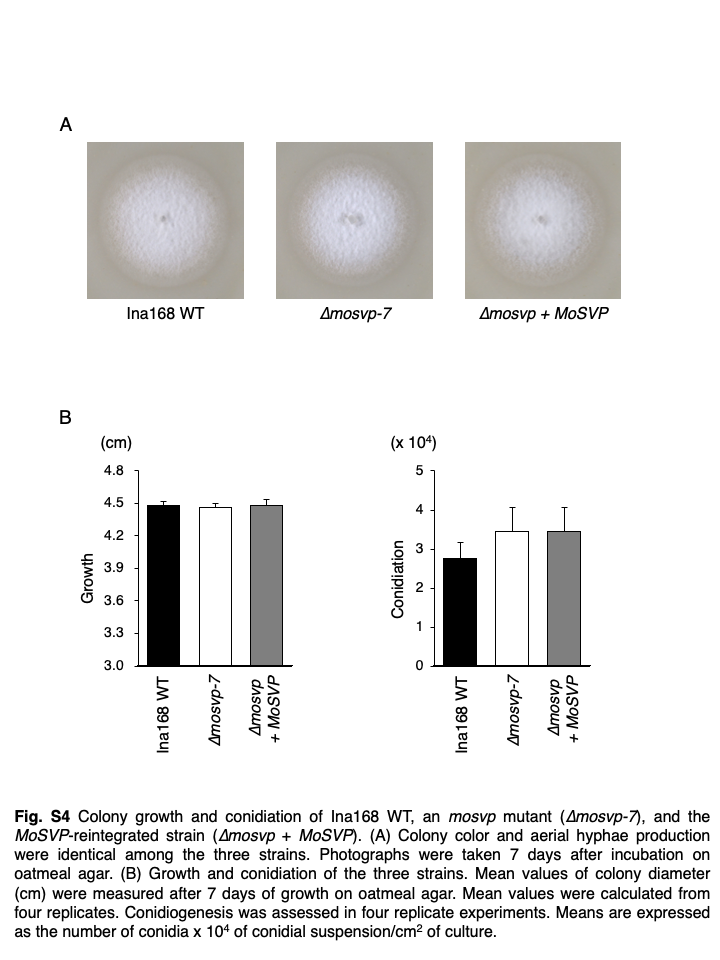

Supplement: Supplementary file 4 — Fig. S4 Colony growth and conidiation of Ina168 wild‐type (WT), an mosvp mutant (∆mosvp‐7), and the MoSVP‐reintegrated strain (∆mosvp + MoSVP). (A) Colony colour and aerial hyphae production were identical among the three strains. Photographs were taken 7 days after incubation on oatmeal agar. (B) Growth and conidiation of the three strains. Mean values of colony diameter (cm) were measured after 7 days of growth on oatmeal agar. Mean values were calculated from four replicates. Conidiogenesis was assessed in four replicate experiments. Means are expressed as the number of conidia × 104 of conidial suspension/cm2 of culture. [file MPP-20-1682-s004.tiff]

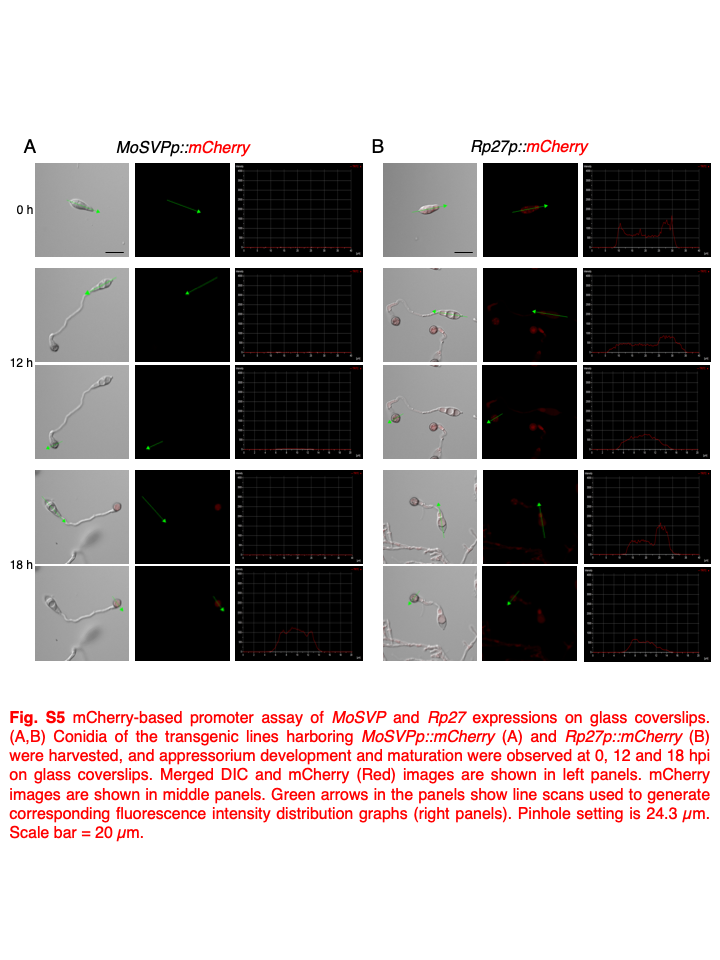

Supplement: Supplementary file 5 — Fig. S5 mCherry‐based promoter assay of MoSVP and Rp27 expressions on glass coverslips. (A,B) Conidia of the transgenic lines harbouring MoSVPp::mCherry (A) and Rp27p::mCherry (B) were harvested, and appressorium development and maturation were observed at 0, 12 and 18 hours post‐inoculation (hpi) on glass coverslips. Merged differential interference contrast (DIC and mCherry (Red) images are shown in left panels. mCherry images are shown in middle panels. Green arrows in the panels show line scans used to generate corresponding fluorescence intensity distribution graphs (right panels). Pinhole setting is 24.3 µm. Scale bar = 20 µm. [file MPP-20-1682-s005.tiff]

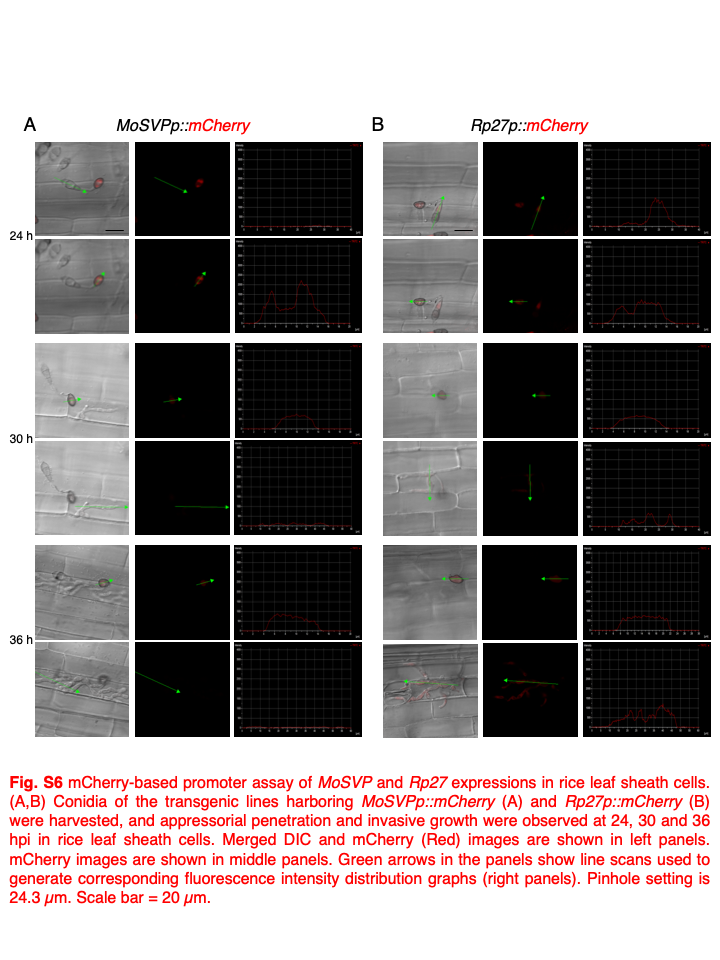

Supplement: Supplementary file 6 — Fig. S6 mCherry‐based promoter assay of MoSVP and Rp27 expression in rice leaf sheath cells. (A,B) Conidia of the transgenic lines harbouring MoSVPp::mCherry (A) and Rp27p::mCherry (B) were harvested, and appressorial penetration and invasive growth were observed at 24, 30 and 36 hours post‐inoculation (hpi) in rice leaf sheath cells. Merged differential interference contrast (DIC) and mCherry (Red) images are shown in left panels. mCherry images are shown in middle panels. Green arrows in the panels show line scans used to generate corresponding fluorescence intensity distribution graphs (right panels). Pinhole setting is 24.3 µm. Scale bar = 20 µm. [file MPP-20-1682-s006.tiff]

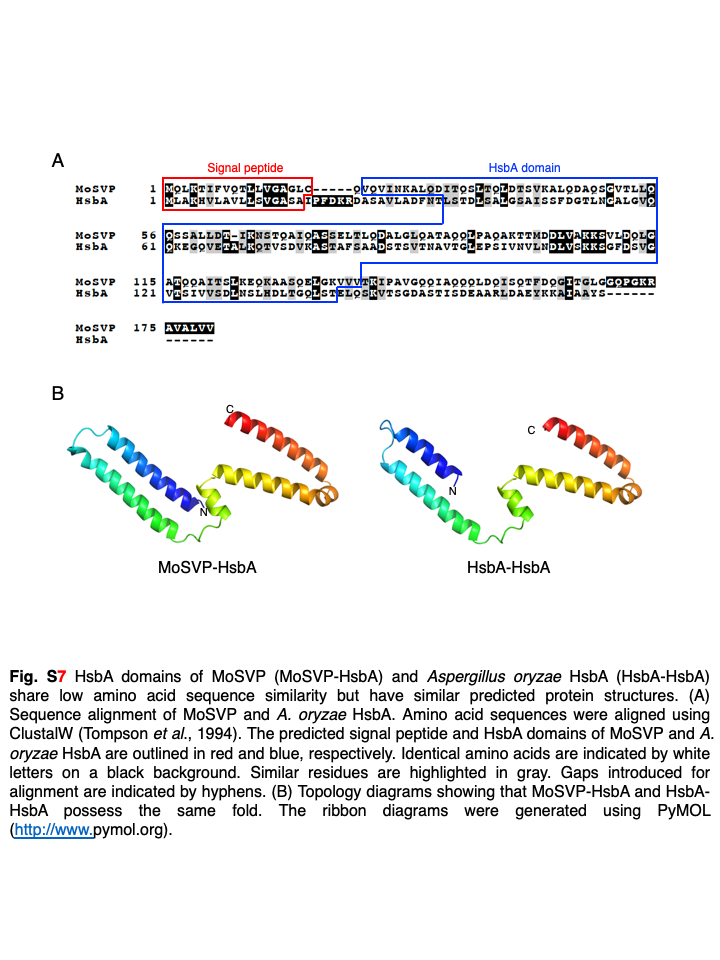

Supplement: Supplementary file 7 — Fig. S7 HsbA domains of MoSVP (MoSVP‐HsbA) and Aspergillus oryzae HsbA (HsbA‐HsbA) share low amino acid sequence similarity but have similar predicted protein structures. (A) Sequence alignment of MoSVP and A. oryzae HsbA. Amino acid sequences were aligned using ClustalW (Tompson et al., 1994). The predicted signal peptide and HsbA domains of MoSVP and A. oryzae HsbA are outlined in red and blue, respectively. Identical amino acids are indicated by white letters on a black background. Similar residues are highlighted in grey. Gaps introduced for alignment are indicated by hyphens. (B) Topology diagrams showing that MoSVP‐HsbA and HsbA‐HsbA possess the same fold. The ribbon diagrams were generated using PyMOL (http://www.pymol.org). [file MPP-20-1682-s007.tiff]

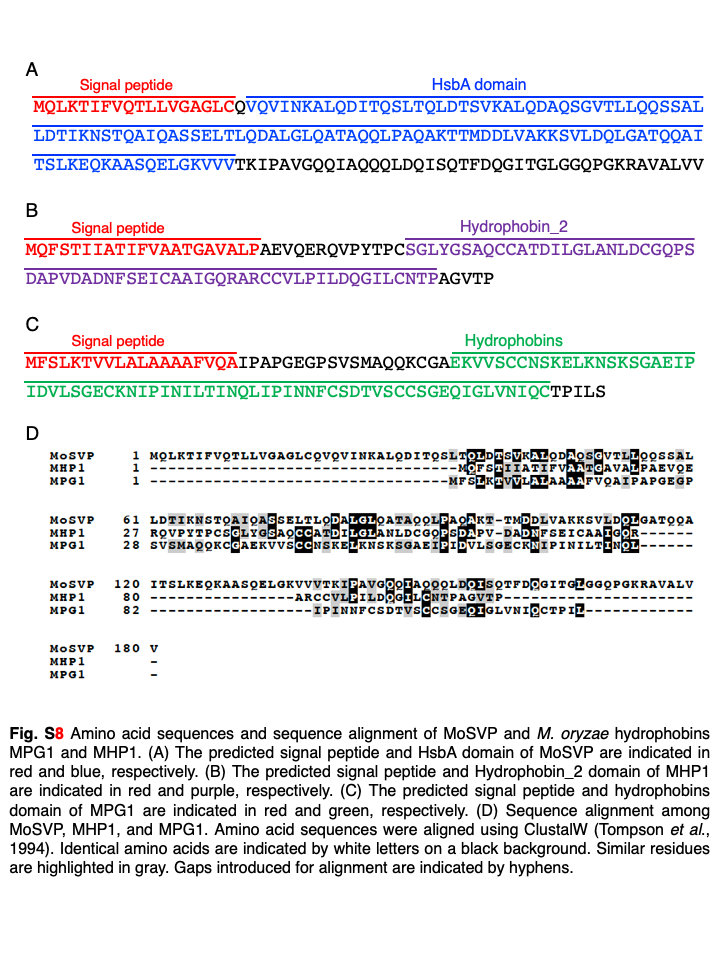

Supplement: Supplementary file 8 — Fig. S8 Amino acid sequences and sequence alignment of MoSVP and Magnaporthe oryzae hydrophobins MPG1 and MHP1. (A) The predicted signal peptide and HsbA domain of MoSVP are indicated in red and blue, respectively. (B) The predicted signal peptide and Hydrophobin_2 domain of MHP1 are indicated in red and purple, respectively. (C) The predicted signal peptide and hydrophobins domain of MPG1 are indicated in red and green, respectively. (D) Sequence alignment among MoSVP, MHP1 and MPG1. Amino acid sequences were aligned using ClustalW (Tompson et al., 1994). Identical amino acids are indicated by white letters on a black background. Similar residues are highlighted in grey. Gaps introduced for alignment are indicated by hyphens. [file MPP-20-1682-s008.tiff]

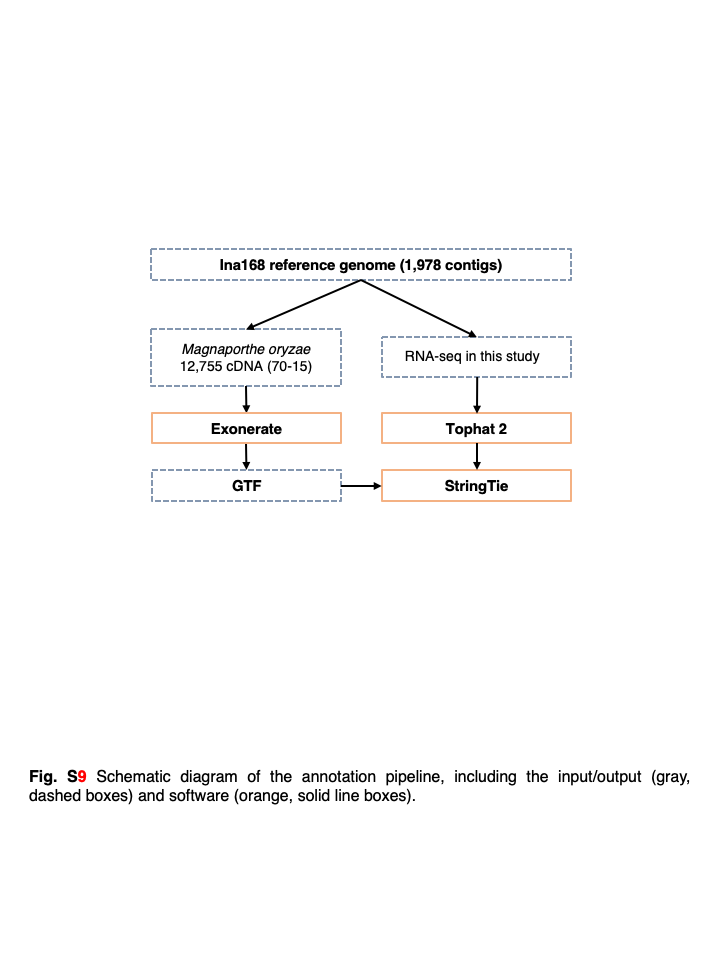

Supplement: Supplementary file 9 — Fig. S9 Schematic diagram of the annotation pipeline, including the input/output (grey, dashed boxes) and software (orange, solid line boxes). [file MPP-20-1682-s009.tiff]
