# Supplementary material for: RNA‐Seq of in planta‐expressed Magnaporthe oryzae genes identifies MoSVP as a highly expressed gene required for pathogenicity at the initial stage of infection
Source: Mol Plant Pathol. 2019 Sep 27;20(12):1682–95. doi: 10.1111/mpp.12869 (PMC6859710; doi:10.1111/mpp.12869)
Supplement: Supplementary file 10 — Table S1 Assembly metrics of the reference genome of Magnaporthe oryzae isolate Ina168. [file MPP-20-1682-s010.pdf]

**Table S1** Assembly metrics of the reference genome of *M. oryzae* isolate Ina168.

| | Strain | |
|---|---|---|
| **Features** | **Ina168** | **70-15** |
| Total reads (Gb) | 1.92 | - |
| Coverage (fold) | 48.4 | - |
| Number of contigs | 1,978 | - |
| Average contigs length (kp) | 20.02 | - |
| Maximum contig length (kp) | 920.1 | - |
| N50 contig length (kb) | 162.9 | - |
| Assembly size (Mb) | 39.61 | 41.03 |
| A | 9,676,511 | 9,901,089 |
| T | 9,666,293 | 9,915,530 |
| G | 10,120,238 | 10,574,756 |
| C | 10,131,235 | 10,558,558 |
| N | 19,978 | 77,800 |
| Assebly size with A/T/G/C only (Mb) | 39.59 | 40.95 |
| G+C composition (%) | 51.1 | 51.6 |

Contigs shorter than 500 bp were excluded.
