# Supplementary material for: RNA‐Seq of in planta‐expressed Magnaporthe oryzae genes identifies MoSVP as a highly expressed gene required for pathogenicity at the initial stage of infection
Source: Mol Plant Pathol. 2019 Sep 27;20(12):1682–95. doi: 10.1111/mpp.12869 (PMC6859710; doi:10.1111/mpp.12869)
Supplement: Supplementary file 11 — Table S2 Coverage of conserved eukaryotic genes in Magnaporthe oryzae isolate Ina168 and 70‐15 genome sequences analysed by CEGMA. [file MPP-20-1682-s011.pdf]

Table S2 Coverage of conserved eukaryotic genes in *M. oryzae* isolate Ina168 and 70-15 genome sequences analyzed by CEGMA.

Strain	Complete*	Partial*
Ina168	236 (95.2%)	242 (97.6%)
70-15	236 (95.2%)	242 (97.6%)

* Number of genes that exhibited a homology to the protein sequences from the 248 core eukaryotic genes with 'complete' and 'partial' coverage for the CEGMA analysis.
