# Supplementary material for: RNA‐Seq of in planta‐expressed Magnaporthe oryzae genes identifies MoSVP as a highly expressed gene required for pathogenicity at the initial stage of infection
Source: Mol Plant Pathol. 2019 Sep 27;20(12):1682–95. doi: 10.1111/mpp.12869 (PMC6859710; doi:10.1111/mpp.12869)
Supplement: Supplementary file 12 — Table S3 Summary of RNA‐Seq reads and mapping statistics. [file MPP-20-1682-s012.pdf]

Table S3 Summary of RNA-seq reads and mapping statistics.

Sample	Replication	[a]Total clean reads	[b]Mapping to Ina168 reference genome	b /a *100 (%)
Conidia	-1	3,787,507	3,712,988	98.0%
	-2	4,278,481	3,927,887	91.8%
	-3	4,566,448	4,354,557	95.4%
12 hpi	-1	21,911,609	205,875	0.9%
	-2	18,863,494	443,718	2.4%
	-3	21,067,292	746,316	3.5%
24 hpi	-1	18,336,408	273,898	1.5%
	-2	16,535,399	454,551	2.7%
	-3	17,890,040	531,192	3.0%
36 hpi	-1	23,380,354	1,427,532	6.1%
	-2	16,869,313	1,225,044	7.3%
	-3	21,487,630	2,579,929	12.0%
48 hpi	-1	11,155,133	2,285,789	20.5%
	-2	10,045,391	3,754,649	37.4%
	-3	9,699,083	2,789,260	28.8%
