# Supplementary material for: RNA‐Seq of in planta‐expressed Magnaporthe oryzae genes identifies MoSVP as a highly expressed gene required for pathogenicity at the initial stage of infection
Source: Mol Plant Pathol. 2019 Sep 27;20(12):1682–95. doi: 10.1111/mpp.12869 (PMC6859710; doi:10.1111/mpp.12869)
Supplement: Supplementary file 14 — Table S5 Primers used for qRT‐PCR and plasmid construction. [file MPP-20-1682-s014.pdf]

Table S5 Primers used for qRT-PCR and plasmid construction.

Primer name	Sequence (5' -3')	Description
MoAct-fwd	TCGACGTCCGAAAGGATCTGT	Real-Time qRT-PCR for *MoAct*
MoAct-rev	GAGCAATGATCTTGACCTTCATCG	
HvUbi-fwd	GCAAGTAAGTGCCTGGTCATGA	Real-Time qRT-PCR for *HvUbi*
HvUbi-rev	ACAACCAGACATGCTCCAACCT	
MoSVPko5fwd	TATAGGGCGAATTGGATTTCGGGTTCAAAAACTGG	Construction of the plasmid pGDMoSVP
MoSVPko5rev	TCGAGGGGGGGCCCGGTTGAAAAAGGGATTTAAAGTT	
MoSVPko3fwd	GAATTCCTGCAGCCCTTTGGTCAAAGGATCCAAAAA	
MoSVPko3rev	GCTGGAGCTCCACCGGTTCGCCAGCTGCCGTCAGTTC	
pCB1636iv1fwd	CGGTGGAGCTCCAGCTTTTGTTCCC	
pCB1636iv1rev	GGGCTGCAGGAATTCGATATCAAGC	
MGG_12247ko5fwd	AATTGGGTACCGGGCTGAATGAGCAAAGACCGCTTTC	Construction of the plasmid for gene disruption of MGG_12247
MGG_12247ko5rev	AACGTCGACCTCGAGATGATCACAAGACTGATGGGTC	
MGG_12247ko3fwd	GAATTCCTGCAGCCCGTGAGGAAGGAGGTGGCAAGTG	
MGG_12247ko3rev	GCTGGAGCTCCACCGGCTTGGCCATCAAGGGATGCTG	
MGG_02253ko5fwd	TATAGGGCGAATTGGGATACCACCAGGAGCAAC	Construction of the plasmid for gene disruption of MGG_02253
MGG_02253ko5rev	TCGAGGGGGGGCCCGTGTGAAAAGAAGTGTGAAGG	
MGG_02253ko3fwd	GAATTCCTGCAGCCCTGAGTGCTTGCGCACTGTCGTC	
MGG_02253ko3rev	GCTGGAGCTCCACCGCGGGTCGTTTTAGGGGTTTTCC	
MGG_05083ko5fwd	AATTGGGTACCGGGCTCTGCACGTGGCGACCAACGTG	Construction of the plasmid for gene disruption of MGG_05083
MGG_05083ko5rev	AACGTCGACCTCGAGGTTTGTTCTCTCAAAGCAAGCG	
MGG_05083ko3fwd	GAATTCCTGCAGCCCCTTTTCTGGGGCCTCGAAAATG	
MGG_05083ko3rev	GCTGGAGCTCCACCGGCCTTCTCCGTTGCCGTCTGTG	
MGG_09351ko5fwd	TATAGGGCGAATTGGGCGTCCAGCTGAACCGCG	Construction of the plasmid for gene disruption of MGG_09351
MGG_09351ko5rev	TCGAGGGGGGGCCCGCTTGAGGCAAGTTGTCGAG	
MGG_09351ko3fwd	GAATTCCTGCAGCCCTGAAAGATGGGGTCATGTTATG	
MGG_09351ko3rev	GCTGGAGCTCCACCGTCGCTGGAACATAACTTAAATC	
MGG_01255ko5fwd	AATTGGGTACCGGGCGGGCCTTTTGTAGAGAATGTAC	Construction of the plasmid for gene disruption of MGG_01255
MGG_01255ko5rev	AACGTCGACCTCGAGCTTTTGTCCTGTCGAGACTGTG	
MGG_01255ko3fwd	GAATTCCTGCAGCCCTGTTGGGCAGTTGGGACTAC	
MGG_01255ko3rev	GCTGGAGCTCCACCGTCCGAATAGGGCGAATACTG	
MGG_16125ko5fwd	TATAGGGCGAATTGGCGCCCGCCGGACCCGCAC	Construction of the plasmid for gene disruption of MGG_16125
MGG_16125ko5rev	TCGACCTCGAGGGGGCTTGATAGCTTTTGAGAAGAT	
MGG_16125ko3fwd	GAATTCCTGCAGCCCCTTCTCTTTTTTTGATTTGGAG	
MGG_16125ko3rev	GCTGGAGCTCCACCGGCGGCCGAAGCAGCTGCGCTTG	
MoSVP_UL	AACGTCGACCTCGAGGCGTAGATTCTCCTTGGATGTC	Construction of the plasmid pCB1531-MoSV
MoSVP_U	AACGTCGACCTCGAGGCGTAGATTCTCCTTGGATGTC	
pCB1636iv2fwd	CTCGAGGTCGACGTTAACTGATATTG	
pCB1636iv2rev	GCCCGGTACCCAATTCGCCCTATAG	
MoSVP-U1	ACCGCGGTGGCGGCCCGCTGAGACTACGAGGAG	Construction of the plasmid pCB-MoSVPp-mCherry
MoSVP-L0	ATCCACTAGTTCTAGAAAAAGGGATTTAAAGTTTTAAAG	
Rp27-U1	ATAAGAATGCGGCCGCATAAATGTAGGTATTACCTGTAC	Construction of the plasmid pCB-Rp27p-mCherry
Rp27-L0	GCTCTAGATTTGAAGATTGGGTTCCTACGAAAG	
